# Does winter desiccation account for seasonal increases in supercooling capacity of Norway spruce bud primordia?

**DOI:** 10.1093/treephys/tpx142

**Published:** 2017-11-22

**Authors:** Edith Kuprian, Sabrina Koch, Caspar Munkler, Anna Resnyak, Othmar Buchner, Marian Oberhammer, Gilbert Neuner

**Affiliations:** University of Innsbruck, Institute of Botany, Unit Functional Plant Biology, Sternwartestr. 15, 6020 Innsbruck, Austria

**Keywords:** extra-organ freezing, freeze dehydration, frost hardiness, ice formation

## Abstract

Bud primordia of *Picea abies* (L.) H. Karst. remain ice free at subzero temperatures by supercooling. Once ice forms inside the primordium, it is immediately injured. Supercooling capacity increases seasonally from ~−5 °C to as much as −50 °C by currently unknown mechanisms. Among other prerequisites, dehydration of tissues over the winter months has been considered to play a key role in freezing tolerance. In this regard, the water content of bud primordia may be crucial, especially in reference to supercooling. In order to assess the role of dehydration in supercooling capacity, seasonal changes in supercooling capacity and the water potential of bud primordia of *Picea abies* (L.) H. Karst were measured at two sites that differed by 1298 m in elevation, after artificial frost hardening and dehardening treatments and after controlled bench drying. The extent of supercooling of bud primordia varied from −7 °C in summer to −24.6 °C in winter, a difference of 17.6 –19.3 K. Total actual water potential (Ψ_t_act__) of bud primordia was −2 MPa in summer and decreased to a mean of −3.8 MPa in midwinter. The decline involved dehydration, and to a lesser extent, osmoregulation. At decreased Ψ_t_act__ values (<3.0 MPa), supercooling capacity significantly increased <−19.5 °C, however, the correlation between actual water potential and supercooling capacity was poor. Frost-hardening treatments increased the supercooling capacity of bud primordia (−0.6 K day^−1^) and lowered Ψ_t_act__ (−0.2 MPa day^−1^). Frost-dehardening treatments reduced supercooling capacity (+1.1 K day^−1^), and at the same time, increased Ψ_t_act__ (+0.3 MPa day^−1^). In contrast, artificial drying of bud primordia in the range observed seasonally (−2.0 MPa) had no effect on supercooling capacity. These results suggest that there is no causal relationship between desiccation and the supercooling capacity of bud primordia in *P. abies*, but rather it involves other compounds within the cells of the bud primordium that reduce the water potential.

## Introduction

Norway spruce (*Picea abies*) bud primordia survive freezing by supercooling and extra-organ ice formation (Pukacki 1987, Buchner and Neuner 2009). These processes also occur in the vegetative buds of many other cold-hardy conifers, with the exception of pines (Sakai 1978, 1979, Sakai and Eiga 1985, Ide et al. 1998), and the reproductive buds of angiosperms (Quamme et al. 1995). After ice formation occurs at subzero temperatures in the apoplast of shoot tissues of *P. abies* (L.) H. Karst, cells lose water to sites of extracellular ice, a process referred to as extracellular freezing. Ice, however, is hindered from propagating into the bud primordium by an efficient ice barrier that is formed by the so-called crown and innermost bud scales (Kuprian et al. 2017). Bud primordia tissues remain completely free of ice and are able to supercool in winter to temperatures as low as −50 °C (Beuker et al. 1998). During freezing with decreasing freezing temperatures, bud primordia become successively freeze dehydrated as temperatures decrease and water migrates across the ice barrier from the bud primordium to a cavity formed inside the pith of the shoot where large extracellular masses of ice develop, a process referred to as extra-organ freezing (Kuprian et al. 2017).

During the course of a year, trees in temperate climate zones undergo genetically controlled frost hardening and dehardening. Shortening photoperiod and episodes of mild frost are the predominant environmental signals triggering cold acclimation and deacclimation (Schwarz 1970). Ambient temperature is an important factor influencing changes in the supercooling capacity of *P. abies* bud primordia (Beuker et al. 1998). Fluctuations in the supercooling capacity of *P. abies* were closely associated with changes in ambient temperature when measured at midwinter of 5 consecutive years (Beuker et al. 1998). In contrast, frost hardening was affected to a much lesser extent by ambient temperature in the autumn, but rather appeared to be much more strongly affected and genetically controlled by photoperiod (Beuker et al. 1998). The supercooling capacity of *P. abies* buds varies between −7 °C at the end of summer (Kuprian et al. 2017) to as low as −50 °C in extremely cold winters (Beuker et al. 1998). The underlying physiological mechanisms regulating changes in the supercooling capacity of bud primordia are not well understood (Wisniewski et al. 2014). Winter adjustments in water relations, related to biochemical and biophysical changes that occur during the course of cold acclimation, have long been considered to play a key role in the frost survival of conifer tissues (Zwiazek et al. 2001, Margesin et al. 2007).

Seasonal changes in the water relations of *P. abies* bud primordia have not been investigated. The water relations of bud primordia can be affected in two ways. First, the overall water content in conifer tissues, in response to low temperatures, has been shown to decrease (Cowling and Kedrowski 1980) with a decrease in the amount of ‘free’ symplastic water and an increase in bound water (Kramer and Kozlowski 1979). Winter desiccation can be much more severe in *P. abies* growing in environmentally extreme sites, such as at or above the alpine timberline, than in other species growing in the same sites (Larcher 1985) and water potentials can drop to as low as −3.5 MPa (Mayr et al. 2012). Osmotic potentials of *P. abies* needles (Ulmer 1937) and shoots (Tyree et al. 1978) were also found to be at a minimum in midwinter. The overall water loss measured in the different organs of *P. abies* should also be reflected in a reduced water content in bud primordia, although this has not been measured or documented.

Secondly, the existence of distantly located masses of ice induces the gradual freeze dehydration of bud primordia, which increases as temperatures decrease. This has been reported to have two major effects, the prevention of intracellular ice formation and an enhancement in the extent of supercooling (Ide et al. 1998). During the controlled freezing of buds of *P. abies* bud primordia become increasingly dehydrated. A drop in water potential up to −2.2 MPa was observed in bud primordia isolated from shoots frozen down to ~−20 °C (Kuprian et al. 2017). In addition, changes in supercooling capacity can also occur in response to ice days, i.e., days where temperature maxima remain subzero, in midwinter (Beuker et al. 1998). These observations indicate that physiological changes responsible for an increase in supercooling capacity can occur when buds are in a frozen state or that freeze dehydration of the supercooled organ per se is sufficient to enhance the supercooling capacity (Ide et al. 1998).

The objective of the present study was to assess the involvement of desiccation in changes in the supercooling capacity of bud primordia of Norway spruce. More specifically, the following questions were investigated: (i) Is the supercooling capacity of bud primordia in *P. abies* related to naturally occurring seasonal changes in water potential? (ii) Do artificial frost hardening and dehardening treatments induce changes in the water potential of bud primordia? (iii) Does controlled dehydration of bud primordia have a corresponding effect on supercooling capacity?

## Materials and methods

### Plant material and study sites

*Picea abies* (L.) H. Karst. is one of the major species of conifer tree inhabiting the boreal zone in Europe and is also predominantly found in montane to subalpine forests in the European Alps. Twigs of *P. abies* bearing several vegetative buds were collected from two sites that differed by 1298 m in elevation. One sampling site was located in the area of the Alpine Garden of the University of Innsbruck on Mt Patscherkofel (1911 m; 47°12′4′N, 11°27′3′E) and the second site was located in the Botanical Garden of the University of Innsbruck (613 m; 47°16′4′N, 11°22′43′E).

Sampling was conducted biweekly between November 2012 and the end of March 2014. Twigs were cut from five 70–90 year old trees at each sampling date from among the natural subalpine population of *P. abies* trees located at the site on Mt Patscherkofel. Samples were stored in a cooling box and transferred to the Institute of Botany within ~1.5 h after collection. Twig samples were taken from five randomly chosen individuals out of 60 potted trees (subalpine ecotypes, 16 years old, obtained from the Landesforstgärten Tirol), that were cultivated outdoors under natural field conditions in the Botanical Garden. Sampled twigs were either used immediately in the experiments or after a period not exceeding 18 h of storage in a cold room (+4 °C).

Measurements were performed on both lateral and terminal buds. In pretests no significant difference in supercooling capacity between buds of different insertion were detected (data not shown). Measurements ceased once buds exhibited signs of budbreak in the spring and commenced again at the end of summer, 2013, when newly formed buds had developed to a size of at least 2 mm.

### Temperature measurements

Air temperature records for the sampling site in the Botanical Garden in Innsbruck were obtained from the ZAMG (Innsbruck University; Central Institute of Meteorology and Geodynamics, Vienna). Air temperatures at the study site on Mt Patscherkofel were recorded throughout the investigation period (mid-November 2012 through the end of March 2014) with a copper-constantan thermocouple (T type, fine-wire, TT-TI40, Omega Engineering Inc., Stamfort, CT, USA) placed 2 m above the ground, close to the investigated *P. abies* trees. Thermocouples were connected to a multiplexer (AM 16/32B, Campbell Scientific, Logan, UT, USA) and temperatures were recorded every minute with a CR1000 data logger (Campbell Scientific).

### Differential thermal analysis

Exothermic events that occur during the freezing of water in plant tissues can be detected and analyzed by differential thermal analysis (DTA). Preparation of Norway spruce buds for DTA measurements was based on the method of Burke et al. (1976). Ten buds attached to a portion of a twig were randomly chosen from the collected shoot samples from each study site and subjected to DTA. Each selected twig was cut in a way that one bud and a 3 cm long piece of the subtending shoot remained. Supernumerous buds were removed with a razor blade; ensuring that any exotherms detected during freezing could be clearly assigned to the residual bud. Differential thermal analysis was conducted using copper-constantan thermocouples (T type, fine-wire, TT-TI40, Omega Engineering Inc., Stamfort, CT, USA) connected to a multiplexer (AM 416, Campbell Scientific). Temperatures were recorded with a data logger (CR10, Campbell Scientific) at a 10 s interval. The thermocouple solder junctions were fixed onto the surface of the outermost bud scales of each investigated bud. The bud and attached thermocouple were wrapped in a thermally conducting, self-adhesive pad (Laird Technologies, Earth City, MO, USA). The bud and surrounding pad were then enveloped in aluminum foil and inserted into wells (6–10 mm in diameter) that had been drilled into aluminum cylinders (diameter 10 cm, height 10 cm). Good thermal contact between the bud sample and the aluminum ensured a high thermal resolution, as even small amounts of exothermic heat were detectable with this experimental setup.

Differential thermal analysis measurements during winter 2012/2013 were performed without application of any ice nucleation active (INA) bacteria (*Pseudomonas syringae* van Hall 1902), as described by Burke et al. (1976). A high-temperature exotherm (HTE), indicative of ice nucleation, was observed in shoots examined during that period of time, between −10.4 and −6.1 °C. This freezing event occurred at a significantly lower temperature than the temperature observed for ice nucleation in woody stems in natural conditions (Neuner and Hacker 2012). Detached plant parts tend to supercool to lower temperatures than attached plant parts (Ashworth et al. 1985, Neuner et al. 1997). This artificial supercooling may lead to an overlap of HTE and low-temperature exotherm (LTE) in twigs that are in a frost dehardened state, making it difficult to separate the two exotherms. Therefore, from mid-July 2013 onwards, ice nucleation in the shoot was triggered by the use of INA bacteria, which allowed freezing to be initiated at moderate freezing temperatures between −1.8 and −3.8 °C (Maki et al. 1974).

**Figure 1.**
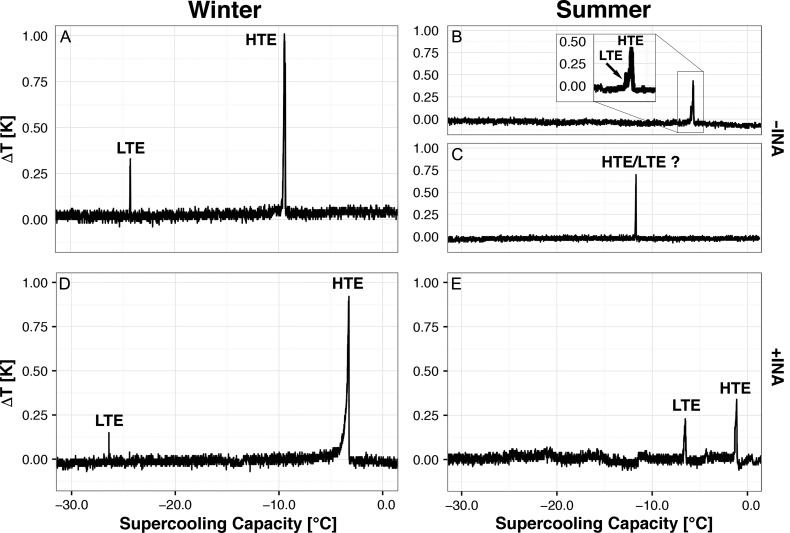
Effect of the use of INA bacteria (*Pseudomonas syringae* van Hall 1902) on the HTE and the detectability of the LTE in *P. abies* bud primordia subjected to DTA. A comparison of DTA-plots obtained without (A–C) and with (D and E) the use of INA bacteria either in winter (A, D) or in summer (B, C, E) shows a clear increase in the temperature at which the HTE is initiated. The HTE and LTE in summer buds can occur at similar temperatures (B) or may even overlap (C), which makes it difficult to distinguish the two freezing events. The use of INA bacteria (E), allows two distinct freezing exotherms to be distinguished even in summer, making precise determination of the HTE and LTE possible.

A unique experimental procedure was developed to administer the INA bacteria to twig samples, as application of single droplets of an INA suspension to the cut shoot surface failed to initiate ice nucleation in the shoot at moderate freezing temperatures (data not shown). Therefore, the following experimental procedure was developed that reliably initiated ice nucleation at ~−2 °C. A sisal cord (30 cm in length) was separated into single fibers and the fibers were then thoroughly soaked with tap water. An ~1 cm longitudinal section was made in the twig sample containing the bud and attached thermocouple, and the sisal fiber was then pinched into the slot made by the incision. This was done prior to the sample being wrapped in the self-adhesive pad and aluminum foil. The sisal fibers were kept wet throughout all the stages of preparation. Shortly before putting the aluminum blocks into a temperature-controlled commercial freezer (Profiline Taurus, National Lab GmbH, Mölln, Germany), the free ends of the sisal fibers were placed into a small container filled with 10 ml suspension of INA bacteria. Crushed ice was added to the tube in order to cool the suspension of INA. In order to ensure that the fibers remained wet during the cooling process, the entire setup (INA suspension and sisal fibers) was wrapped with wet paper towels and plastic film when the samples were placed in the freezer. As evidenced by an HTE, this setup reliably induced the initial freezing of the shoot samples, at temperatures around −2.9 °C (Figure 1), as occurs in nature. This was particularly important as otherwise the LTE would not have been descernable in twigs in a frost dehardened state.

After preparation, the twig/bud samples were subjected to controlled freezing inside a computer-controlled (software developed by O. Buchner in LabView 2012, National Instruments Corporation, Austin, TX, USA) commercial freezer (see Hacker and Neuner 2007). After an equilibrium phase, where samples were maintained at +5 °C for 45 min, the samples were cooled at a rate of 3 K h^−1^ down to a target temperature. The target temperature was determined and set to ensure the killing of the bud primordia. This low-temperature set point varied between −40 and −60 °C from summer to winter.

### Infrared differential thermal analysis

Freezing processes and ice propagation in plant tissues can be localized and visualized by infrared differential thermal analysis (IDTA) (Neuner and Kuprian 2014). Using a digital infrared camera (ThermaCAM S60, FLIR Systems, Danderyd, Sweden) freezing of plant samples can be monitored. An experimental setup was designed to allow for the study of the freezing process in shoots and bud primordia of *P. abies* and at the same time allow the use of attached twigs to measure regrowth capacity after controlled freezing treatments. A branch bearing one terminal and several lateral buds, still attached to a potted *P. abies* tree, was enclosed inside an exposure chamber of a Low Temperature Freezing System (LTFS). The LTFS allows controlled in situ freezing treatments down to −70 °C to be conducted on attached twigs (Buchner and Neuner 2009). Four thermocouples were fixed near the buds and the ambient temperature of the exposure chamber was collected every 10 s with a data logger (CR10X, Campbell Scientific). The branch was mounted in a manner that allowed it to be viewed with the digital infrared camera from outside during controlled freezing through an infrared permeable (0.45–12 μm) inspection window (10 × 10 × 0.5 cm^3^ glass made of zinc oxide, ZnS clear grade; Vitron, Jena-Maua, Germany). Dry air was continuously blown onto the window surface to prevent condensation of water on the inspection window from interfering with the accurate measurement of the twig sample temperature by IDTA. The infrared camera was equipped with a close-up lens (LW64/150) to achieve a spatial resolution of 200 μm. Infrared images were recorded at a measurement interval of 100 ms. Further analysis of the infrared images was performed with ThermaCAM Researcher (FLIR Systems, Danderyd, Sweden) software. Ice nucleation events were detected using an IDTA protocol (Hacker and Neuner 2007, 2008, Hacker et al. 2008, Neuner and Kuprian 2014).

### Artificial dehydration treatment

Detached *P. abies* twigs (7 cm in length), bearing lateral and terminal vegetative buds, were exposed to room temperature (+20 °C) without water for 1–4 days under moderate illumination (~40 μmol photons m^−2^ s^−1^). After the selected time span, total water potential (Ψ_t_act__) was assessed and then the supercooling capacity of bud primordia was determined by DTA.

### Artificial frost hardening and dehardening

Sampled twigs of *P. abies* bearing numerous vegetative buds were put in a bucket filled with tap water in order to ensure a continuous adequate water supply to the twigs. Frost-hardening treatment: the bucket with the sampled twigs was placed inside a temperature-controlled commercial freezer (GTS 2112, Liebherr, Austria) and kept at a constant −6 °C in darkness.

Frost-dehardening treatment: another set of sampled *P. abies* twigs was put into a water-filled bucket and the twigs were collectively wrapped in a plastic bag to minimize transpiration. The bucket with the wrapped twigs was then placed in a greenhouse at the Botanical Garden of the University of Innsbruck, with a daytime temperature of +15 °C and a night minimum of +10 °C under natural daylight and photoperiod.

### Total actual water potential (Ψ_t_act__)

The measurement of the total actual water potential (Ψ_t_act__) of bud primordia of detached vegetative buds of *P. abies* was conducted using eight C-52 sample chambers (Wescor Inc., Logan, UT, USA), which were connected to a PSYPRO water potential system (Wescor Inc.). An RS-232 interface and the PSYPRO software enabled communication between the PSYPRO and a personal computer. The device measures water potentials based on psychrometric records. The chambers used sample holders with a diameter of 7 mm and a depth of 2.5 mm. NaCl solutions (OPTI-MOLE^TM^ Osmolality Standards, manufactured for Wescor, Inc.) with concentrations of 100, 290 and 1000 mmol kg^−1^ were used for calibration. The standards were applied to 6 mm diameter filter paper discs (Whatman, Schleicher & Schuell, type 597, pore size 4–7 μm, Little Chalfont, UK). The total water potential of whole, isolated bud primordia was measured. Buds were removed from the subtending shoot with a razor blade in order to obtain the total water potential of only the primordia. Subsequently, thin transverse sections were cut and removed, starting at the base of the bud (Lewis and Dowding 1924, Cesar and Bornman 1996) until the almost transparent cell layers of the apical bud tissue became visible. At this point, the bud scales were removed with a gentle twisting and squeezing movement. The isolated bud primordium was then immediately placed inside the sample holder of a C-52 chamber for water potential measurement.

### Osmotic water potentials (Ψ_o_)

Osmotic water potentials were obtained on samples of cell sap expressed from *P. abies* bud primordia as described in detail by Kikuta and Richter (1992). Samples were taken at monthly intervals at the subalpine site from January 2008 through April 2009. Expressed saps for the determination of actual osmotic water potentials (Ψ_o_act__) were obtained from bud primordia immediately after sampling at the subalpine site in the Alpine Garden Laboratory of the University of Innsbruck. Osmotic water potentials at full saturation (Ψ_o_sat__) were determined after twigs had been saturated with water. To saturate the twigs, the sampled twigs were immediately placed in a bucket of water after sampling, covered with a plastic bag and placed at room temperature for 24 h. Buds from the saturated twigs were then sampled and prepared as described to measure Ψ_o_sat__.

### Statistical analysis

Mean values of daily maximum and minimum air temperatures were calculated for each study site from November 2012 to the end of March 2014. A two-way, fixed-factor analysis of variance (GLM) was applied to test the effects season and site on supercooling capacity (LTE) of *P. abies* bud primordia (IBM SPSS Statistics for Windows, Version 21.0. Armonk, NY, USA). A significance level of *P* ≤ 0.01 was used for all statistical tests.

## Results

### Freezing response of bud primordia

**Figure 2.**
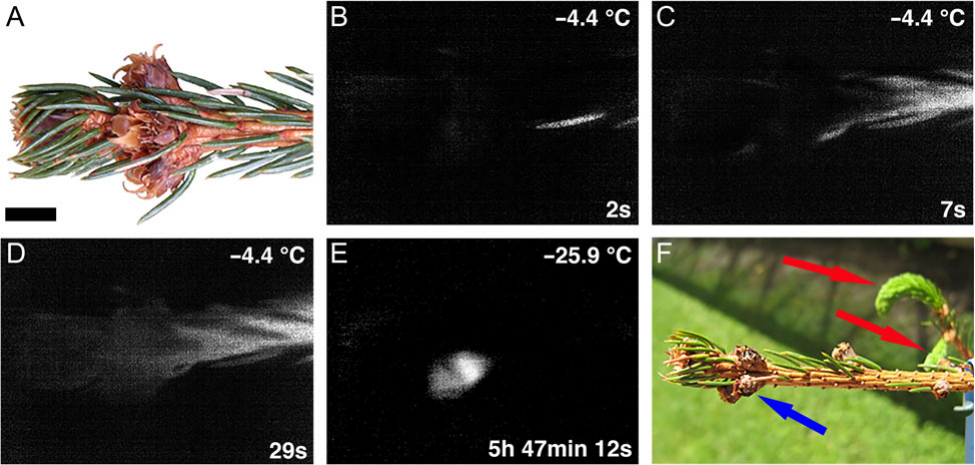
Images of a *P. abies* twig before (A), during (B–E) and 4 months after (F) a controlled freezing experiment. The attached twig was cooled at a rate of 4 K h^−1^. (B) Initial ice nucleation occurred in the stem at ~−4.4 °C and propagated throughout the whole shoot and attached needles (C, D). Bud primordia froze individually and at significantly lower freezing temperatures. The freezing pattern (E) of a single bud primordium at −25.9 °C during the LTE is shown. (F) After the freezing treatment, the ability of the buds to regrow was monitored. While untreated buds burst in springtime and developed new shoot and needles (red arrows), the freeze-treated buds did not sprout (blue arrow) as they were freeze killed by the freezing treatment. Actual temperatures are indicated in the top right corner of each image. The time span (in hours, minutes and seconds) after initial ice nucleation in the shoot is indicated at the bottom right corner. Horizontal black bar = 0.5 cm.

Two freezing exotherms were typically recorded in buds of *P. abies* (Figure 1A) during the winter months. A mean HTE of −8.4 ± 0.2 °C was detected when INA bacteria were not used to induce ice nucleation. Low-temperature exotherms were always characterized by brief single-peaked freezing exotherms recorded at much lower freezing temperatures (−24.4 ± 0.2 °C) than HTEs in the winter months. Without the application of INA bacteria the LTE in summer months occurred at temperatures very close to the HTE (Figure 1B), which often made the distinction between the two events problematic or impossible (Figure 1C). The use of INA bacteria, as described in the Materials and methods, however, induced the HTE to occur at a 5.5 K warmer temperature, around −2.9 °C (Figure 1D). This enabled the separation of the two exotherms and a clear discrimination of the LTE from HTE when the supercooling capacity of bud primordia was at its minimum (Figure 1E). The mean LTE in August and September occurred at −5.3 ± 1.3 °C (data from lowland site; Table 1). The pattern of ice propagation around *P. abies* buds was monitored by IDTA in an attached twig of a potted tree in February (Figure 2A–C). Images reveal that the HTE results from the freezing of the shoot, needles and bud scales and that the LTE originates from freezing of a bud primordium. All bud primordia in which LTEs were detected were dead, as determined by their inability to break bud and regrow in spring (Figure 2F).

**Table 1.** Mean minimum and mean maximum temperature (°C) recorded at the two study sites and respective mean, minimum and maximum supercooling capacity (LTE, °C) and its seasonal amplitude (∆LTE) at the investigation sites.

	$\overset{¯}{T_{\mathrm{Min}}}\cdot ({}^{\circ}C)$	$\overset{¯}{T_{\mathrm{Max}}}\cdot ({}^{\circ}C)$		LTE (°C)			
				Mean ± SE	Max	Min	Seasonal ∆LTE
Subalpine site (mountain; 1911 m)	−1.5	+8.0	Summer	−7.2 ± 0.9	−18.9	−2.1	17.4
			Winter	−24.6 ± 0.3	−28.6	−18.5	
Lowland site (valley; 613 m)	+4.9	+15.3	Summer	−5.3 ± 1.3	−14.6	−1.1	19.3
			Winter	−24.6 ± 0.2	−27.7	−20.7	

### Seasonal changes in supercooling capacity and water potential of bud primordia

**Figure 3.**
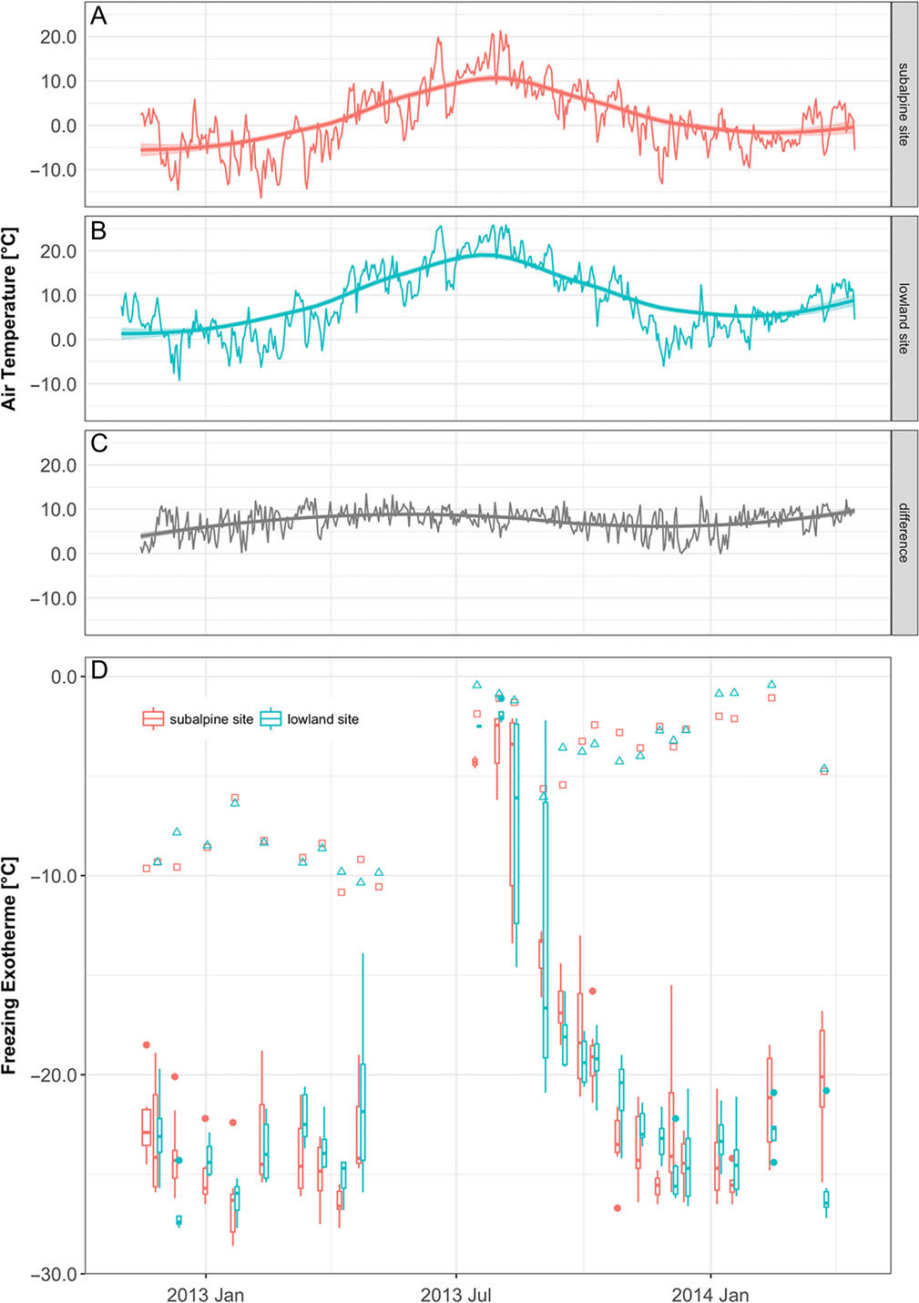
(A) Daily mean air temperature at the subalpine site on Mt Patscherkofel (1911 m) and (B) at the lowland site (613 m) throughout the investigation period. (C) Daily temperature differences between mean temperature at the lowland and subalpine site, respectively. (D) Mean temperature of the HTE in *P. abies* shoots is indicated by red lines (subalpine site) and blue lines (lowland site). The box plots show the variation in the LTEs, i.e., the supercooling capacity of *P. abies* bud primordia. The line inside the box is the median and the box extends from the first to the third quartile. The whiskers extend at maximum to the 1.5-fold interquartile range. Outliers are shown as dots.

The supercooling capacity of *P. abies* bud primordia was monitored under similar photoperiod but contrasting ambient air temperature conditions at two sites that differed in elevation by 1298 m (Figure 3). As expected, the subalpine site was significantly colder than the lowland site. Respective mean minimum and maximum temperatures were 6.4 K and 7.3 K lower at the subalpine site than at the lowland site (Table 1). Despite the contrasting ambient temperatures, the LTEs detected in bud primorida from the two sites were not significantly different (Table 2). Midwinter supercooling capacity was −24.6 °C and similar at both sites. The mean LTE at the subalpine site was −7.2 ± 0.9 °C in the summer, which was 1.9 K lower than at the lowland site. Significant seasonal changes in the range of 17.4 K at the subalpine site and 19.3 K at the lowland site were observed. For samples collected in the autumn, no significant differences in the temperature at which the LTEs occurred were observed between the two sites.

**Table 2.** Results of a two-way fixed-factor analysis of variance (GLM) of the effects of ‘season’ (seasonal period) and ‘site’ (subalpine or lowland) on supercooling capacity of bud primordia of *P. abies* (LTE). Significant effects are given in bold. Abbreviation: *P*, error probability; significance level: 0.01.

Supercooling capacity (LTE)	
Source of variation	*P*
Season (Se)	**<0.001**
Site (Si)	0.490
Se * Si	<**0.001**
*R*^2^	0.653

**Figure 4.**
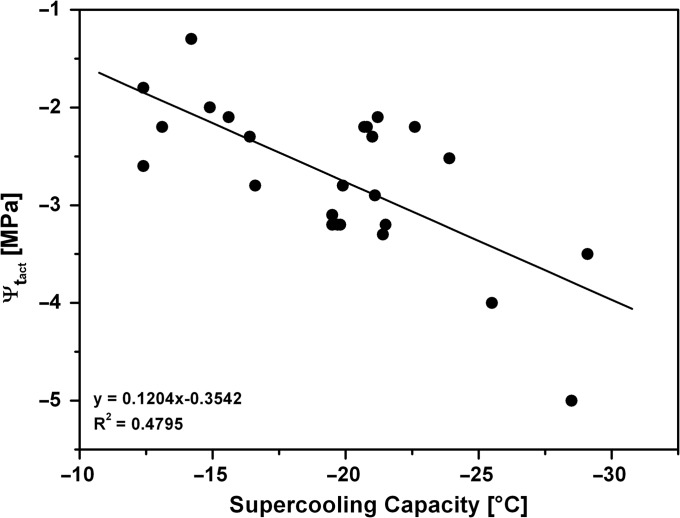
Linear correlation between the supercooling capacity (LTE, °C) of *P. abies* bud primordia and their actual total water potential (Ψ_t_act__).

**Figure 5.**
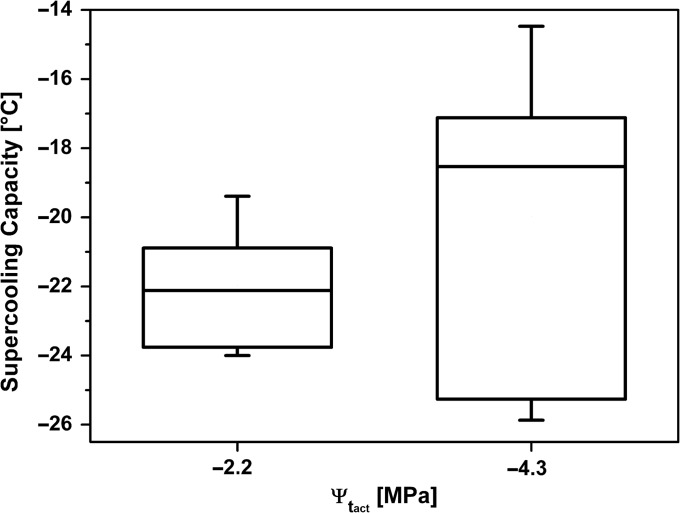
Supercooling capacity (LTE, °C) of *P. abies* bud primordia with a mean water potential (Ψ_t_act__) of −2.2 and −4.3 MPa before and after controlled bench drying, respectively.

Total actual water potential (Ψ_t_act__) of *P. abies* bud primordia decreased significantly during the winter months. The lowest water potential for *P. abies* bud primordia recorded under natural frost hardening in midwinter (2 February 2014) was –5.0 MPa (mean −3.8 MPa). On the other hand, during summer, Ψ_t_act__ values were typically close to −2 MPa. The supercooling capacity of bud primordia distinctly increased at Ψ_t_act__ values lower than −3.0 MPa (Figure 4). Low-temperature exotherms at these low water potentials were recorded at temperatures below −19.5 °C, however the linear correlation between LTEs and Ψ_t_act__ was weak (*R*^2^ = 0.47956; Figure 5).

The mean seasonal decrease of Ψ_t_act__ by −1.8 MPa in *P. abies* bud primordia appears to originate from dehydration. In seasonally determined osmotic water potentials (Table 3), actual osmotic water potential (Ψ_o_act__) was −1.0 MPa lower than the osmotic water potential at full saturation, Ψ_o_sat__. To a lesser extent, osmoregulation, detected as a lowering of Ψ_o_sat__, is involved in the reduction of water potentials of bud primordia in winter. Although not significant under the experimental conditions, the mean value of Ψ_o_sat__ was lowered in winter (−3.0 ± 0.4 MPa) as compared with summer (−2.3 ± 0.5 MPa).

**Table 3.** Seasonal changes in actual osmotic water potential Ψ_o_act__ (MPa) and osmotic water potential at full saturation Ψ_o_sat__ (MPa) of bud primordia of *P. abies* measured at the subalpine site from Januray 2008 till April 2009. Differences between mean values were not significant, which was tested by ANOVA and Duncan Multiple Range Test (significance level: 0.05).

Season (months)	Ψ_o_act__ (MPa) mean ± SE (*N*)	Ψ_o_sat__ (MPa) mean ± SE (*N*)
Autumn (10–11)	−3.2 ± 0.2 (10)	−2.6 ± 0.1 (10)
Winter (12–3)	−4.0 ± 0.5 (8)	−3.0 ± 0.4 (8)
Spring (4–5)	−3.3 ± 0.4 (9)	−2.6 ± 0.3 (9)
Summer (6–9)	−3.1 ± 0.5 (5)	−2.3 ± 0.5 (4)

### Effect of frost hardening and dehardening treatments on LTE and water potential

**Figure 6.**
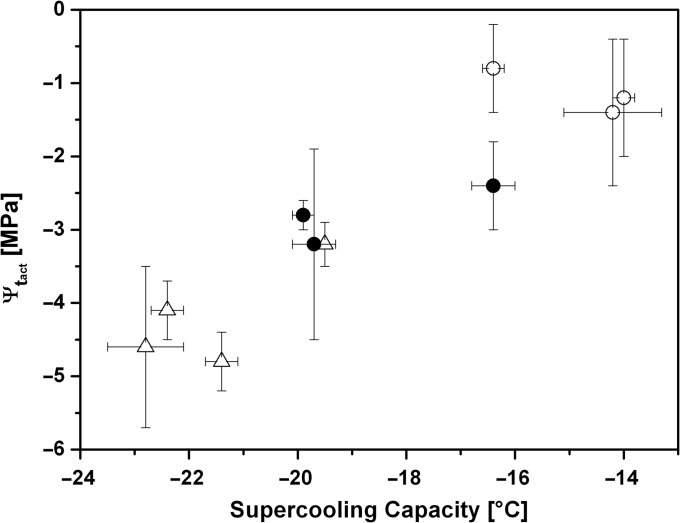
Mean values ± SE of the supercooling capacity (LTE, °C) and actual total water potential (Ψ_t_act__) of *P. abies* bud primordia at the end of April 2015 before (●) and after controlled artificial frost hardening (○) and dehardening (∆) treatments.

*Picea abies* bud primordia collected at the end of April 2015 were exposed to temperature treatments designed to induce frost hardening (exposure to −6 °C) or frost dehardening (exposure to +16 °C). The frost-hardening treatment increased supercooling capacity and lowered Ψ_t_act__, while the frost-dehardening treatment reduced the supercooling capacity of the bud primordia and also increased Ψ_t_act__ (Figure 6).

Within 10 days of exposure to the frost-hardening treatment, the supercooling capacity of bud primordia changed by −4 K, increasing from −19.8 to −23.8 °C, at a rate of −0.4 K day^–1^. During the frost-hardening treatment, the total water potential of bud primordia dropped from −3.2 MPa to ~−4.8 MPa, decreasing at a rate of −0.3 MPa day^−1^. In response to the frost-dehardening treatment, the LTE decreased by +5.8 K within 4 days, from a mean of −19.8 °C to a mean of −14.0 °C, at a rate of +1.9 K day^−1^. Water potential became successively less negative and increased from −3.2 MPa to around −1.5 MPa, at a rate of +0.4 MPa day^−1^.

Similar frost hardening and dehardening treatments were also administered in March and May. Overall, frost-hardening treatments induced an increase in supercooling capacity at a rate of −0.6 K day^−1^ concomitant with a decrease in water potential of −0.2 MPa day^−1^. Frost-dehardening treatments induced a decrease in the mean LTE at a rate of +1.1 K day^−1^ and an increase in water potential of +0.3 MPa day^−1^.

### Effect of artificial dehydration on supercooling capacity

*Picea abies* twigs bearing bud primordia were subjected to a controlled drying procedure (+20 °C) on a lab bench in November and December 2015, in which the water potential of bud primordia was artificially reduced by −2 MPa. A water potential of −2 MPa comes close to the naturally observed seasonal water potential amplitude. Bud primordia had a mean Ψ_t_act__ of −2.2 MPa prior to the start of the desiccation treatment, and an LTE at −22.0 ± 0.4 °C (Figure 6). When bud primordia exhibited a mean Ψ_t_act__ of −4.3 MPa, which required up to 4 days of bench drying, buds were subjected to DTA and exhibited an LTE of −20.0 ± 1.6 °C. Despite the significant changes in Ψ_t_act__, no change in supercooling capacity was observed.

## Discussion

### Effect of water potential on supercooling capacity of *P*. *abies* bud primordia

While artificial dehydration (bench drying) of *P. abies* bud primordia did not affect their supercooling capacity, artificial frost-hardening and frost-dehardening treatments induced a respective decrease or increase in water potential of bud primordia with a concomitant increase or decrease in supercooling capacity. A similar relationship was observed in bud primordia exposed to natural hardening and dehardening conditions over the course of the year. Total water potential of a tissue is determined by water content. Therefore, dehydration will decrease Ψ_t_, but an osmotic component (Ψ_o_) and cell wall turgor pressure (Ψ_p_) also are contributing factors. Our results indicate that dehydration per se does not affect the supercooling capacity of bud primordia. Thus, the two other potentially relevant components of total water potential must be considered.

More negative osmotic potentials result from increased solute concentration, brought about by either by dehydration (water loss) or the accumulation of solutes. Thus, only osmotic potentials determined after full water saturation Ψ_o_sat__ are indicative of osmoregulation, i.e., changes in solute content (Lösch 1993). The Ψ_o_sat__ values of *P. abies* bud primordia decreased by 0.7 MPa in winter, but under experimental conditions, the decrease was not significant. Decreased Ψ_o_sat__ values have been repeatedly found in leaves of woody plants during winter, such as *Picea glauca* (Colombo and Teng 1992) and *Rhododenrdon ferrugineum* (Neuner et al. 1999). The Ψ_o_sat__ of *P. abies* shoots reached a minimum in midwinter at the time of maximum cold hardiness (Tyree et al. 1978). During winter, the maximum extent of the decrease in Ψ_o_sat__ in leaves is close to −1.5 MPa (Colombo and Teng 1992, Neuner et al. 1999). Osmoregulation is generally thought to improve the dehydration tolerance of cells (Lösch 1993) and therefore must also be considered as an important effect during freeze dehydration.

While an osmotic adjustment over winter has already been reported for evergreen conifer shoots and leaves of other evergreen tree species (see Pramsohler and Neuner 2013), little is known regarding seasonal adjustments in the water relations of bud primordia. In contrast to *P. abies* buds, apple bud primordia do not exhibit supercooling but rather undergo freeze dehydration losing cellular water to sites of ice outside of the primordium. Interestingly, Ψ_o_sat__ values of apple terminal bud tissue exhibit no significant seasonal changes, even though values tended to increase slightly in spring and a minor level of dehydration occurred in winter as determined by reduced Ψ_o_sat__ (Pramsohler and Neuner 2013). The same situation appears to exist in *P. abies* bud primordia, although due to a completely different frost survival mechanism. Potential solute candidates that could lower Ψ_o_sat__ in *P. abies* primordia are di-, tri- and tetrasaccharides. These solutes have been recently demonstrated to accumulate in *P. abies* primordia, close to the tissue making up the ice barrier that exists in winter (Kuprian et al. 2017).

A lesser cell wall turgor pressure (Ψ_p_), resulting from alterations in cell wall elasticity, could also reduce Ψ_t_ during winter. Seasonal changes in the composition of cell walls of bud primordia could also be possible, however cells in the primordia are composed of undifferentiated cells with only primary cell walls that are thin and not fully developed with potentially little leeway for changes in elasticity. Based on our data (midwinter mean of Ψ_o_act__ of −4.0 and Ψ_t_act__ of −3.8 MPa) it appears that Ψ_p_ amounts to ~0.2 MPa in bud primordia, and as a result, must be considered as only a minor component having little effect on Ψ_t_act__.

During a freezing event, freeze dehydration significantly lowers the water potential of bud primordia. In bud primordia collected in winter, controlled freezing down to a sublethal temperature of −24 °C reduced Ψ_t_act__ from an initial −3.5 MPa to −5.7 MPa, representing a reduction of −2.2 MPa (Kuprian et al. 2017). Dehydration of the shoot apex and needle primordia has been suggested to have two major effects: prevention of intracellular ice formation and an enhancement in supercooling capacity (Ide et al. 1998). Our results, however, do not fully support this view as dehydration per se had no significant effect on the supercooling capacity of *P. abies* bud primordia. Dehydration, however, may still be important during freezing when other frost resistance mechanisms are in place and active. Reduced water content in winter decreases the size and/or amount of ice masses that have to be accommodated in the tissue and thus would lower the consequent injury resulting from mechanical disruption of cells by the growing ice masses.

### Seasonal changes in the supercooling capacity of Norway spruce buds

At the subalpine and lowland site, the mean supercooling capacity of Norway spruce buds in August was determined to be −7.0 and −5.6 °C, respectively. The mean values decreased in September to −15.9 and −16.2 °C at the subalpine and lowland sites, respectively. These data corroborate earlier results of a comprehensive study on buds of four provenances of *P. abies* tested over 5 consecutive years where a supercooling capacity ranged between −8 and −20 °C (Beuker et al. 1998). The freezing process in *P. abies* buds produces two distinct freezing exotherms. An LTE, which reflects the lethal freezing of intracellular water in cells of the primordium, could only be unambiguously detected when INA bacteria were used to initiate freezing at a warm subzero temperature. Without the use of INA bacteria, as was done in the initial studies, initial freezing, as indicated by an HTE, was observed between −8 and −16 °C (Pukacki 1987); this is clearly in the range of occurrence of an LTE in samples collected in the summer months. The presence of INA bacteria induces an HTE in the shoot at −2.9 °C, close to the ice nucleation temperature of trees in nature (Neuner and Hacker 2012). Avoidance of artificial supercooling is extremely important for determining the presence of an LTE, especially in summer buds. An artificially steep water potential gradient would be induced in bud primordial cells that could potentially influence the analysis of supercooling capacity.

The mean supercooling capacity of *P. abies* bud primordia in winter was −24.6 °C at both sites. The midwinter supercooling capacity observed in our study was is in the range reported for buds of northern European provenances of *P. abies*, which were −27.0 °C (Pukacki 1987) and −20 to −50 °C (Beuker et al. 1998). Beuker et al. (1998) found no significant difference in midwinter supercooling capacity of buds collected from trees of different provenances cultivated at the same site. If any changes in midwinter supercooling capacity were observed, they occurred rapidly within a 1 week period and were highly correlated with changes in daily minimum temperatures (Beuker et al. 1998). Interestingly, Beuker et al. (1998) found that genetic differences between provenances were only visible during natural frost hardening in the fall. The results of the present study indicate that differences in air temperature minima of −6.4 K were not effective in triggering an increase in supercooling capacity at the subalpine site. This may indicate that the critical freezing temperature thresholds needed for the induction of additional frost hardening of bud primordia did not occur at either the lowland or the subalpine site. The bud primordia at both sites may have frost hardened only to the necessary winter maxima present during the period of investigation, which were −27.7 and −28.6 °C at the lowland site and at the subalpine site, respectively, even though the supercooling capacity of *P. abies* bud primordia has been reported down to ~−50 °C (Beuker et al. 1998).

## Conflict of interest

None declared.

## Funding

Support for this research project came from the Austrian Science Fund (FWF). The project P23681-B16 was granted to G.N., giving financial support for the PhD scholarship of E.K.
